# Combined statistical modeling enables accurate mining of circadian transcription

**DOI:** 10.1093/nargab/lqab031

**Published:** 2021-04-26

**Authors:** Andrea Rubio-Ponce, Iván Ballesteros, Juan A Quintana, Guiomar Solanas, Salvador A Benitah, Andrés Hidalgo, Fátima Sánchez-Cabo

**Affiliations:** Area of Cell and Developmental Biology, Centro Nacional de Investigaciones Cardiovasculares (CNIC), Madrid 28029, Spain; Bioinformatics Unit, Centro Nacional de Investigaciones Cardiovasculares (CNIC), Madrid 28029, Spain; Area of Cell and Developmental Biology, Centro Nacional de Investigaciones Cardiovasculares (CNIC), Madrid 28029, Spain; Area of Cell and Developmental Biology, Centro Nacional de Investigaciones Cardiovasculares (CNIC), Madrid 28029, Spain; Institute for Research in Biomedicine (IRB Barcelona), The Barcelona Institute of Science and Technology (BIST), Barcelona 08028, Spain; Catalan Institution for Research and Advanced Studies (ICREA), Barcelona 08010, Spain; Institute for Research in Biomedicine (IRB Barcelona), The Barcelona Institute of Science and Technology (BIST), Barcelona 08028, Spain; Catalan Institution for Research and Advanced Studies (ICREA), Barcelona 08010, Spain; Area of Cell and Developmental Biology, Centro Nacional de Investigaciones Cardiovasculares (CNIC), Madrid 28029, Spain; Bioinformatics Unit, Centro Nacional de Investigaciones Cardiovasculares (CNIC), Madrid 28029, Spain

## Abstract

Circadian-regulated genes are essential for tissue homeostasis and organismal function, and are therefore common targets of scrutiny. Detection of rhythmic genes using current analytical tools requires exhaustive sampling, a demand that is costly and raises ethical concerns, making it unfeasible in certain mammalian systems. Several non-parametric methods have been commonly used to analyze short-term (24 h) circadian data, such as JTK_cycle and MetaCycle. However, algorithm performance varies greatly depending on various biological and technical factors. Here, we present CircaN, an *ad-hoc* implementation of a non-linear mixed model for the identification of circadian genes in all types of omics data. Based on the variable but complementary results obtained through several biological and *in silico* datasets, we propose a combined approach of CircaN and non-parametric models to dramatically improve the number of circadian genes detected, without affecting accuracy. We also introduce an R package to make this approach available to the community.

## INTRODUCTION

Circadian rhythms are cycles in biological processes that last ∼24 h (*circa diem*). They have been described from bacteria to primates (1), and have a profound impact in a wide range of physiological processes, including body temperature, active/resting periods, hormonal regulation, immunity and even mood states (2,3). It is therefore not surprising that circadian gene expression has been the object of great interest and scrutiny, especially since the rise of technologies that have popularized whole transcriptomic analysis.

Immune and epidermal stem cells, as well as organs, are among the many biological systems in which circadian oscillations have been scrutinized. These studies have revealed cell- and tissue-specific patterns of gene expression featuring circadian patterns (4–7). Detection of circadian genes in each system has in turn been critical to unveil key mechanisms of tissue physiology that operate in healthy individuals, and become aberrant during aging or disease (4,7–9). To accurately identify true circadian transcriptional oscillations, it is imperative to sample the system under study with high frequency (e.g. every hour) and for extended periods of time, typically 48 h in practical scenarios. This intense sampling, however, is usually only feasible for non-mammalian organisms, such as flies, or for cells in culture, in which samples are abundant and simple to collect. When dealing with complex mammalian systems, however, repeated sampling can be unfeasible given the cost and difficulty to access certain genetic models or species. A common approach to deal with the limited number of animals is to space sampling frequency to every 2–6 h (7,10–12). While this may not be a problem for 48 h long experiments, sampling frequencies longer than 2 h in 24 h long experiments make it difficult to infer infradian or ultradian genes (5), and complicate the identification of circadian genes. An additional effect of limited experimental samples is the need to reduce the number of biological replicates per time point, which greatly increases noise.

These experimental limitations demand robust statistical methods to identify circadian genes and proteins in biological settings, particularly in mammalian systems. Two popular algorithms are mainly used to analyze circadian experiments; the first is JTK_cycle (herein JTK) (13), which applies the non-parametric Jonckheere–Terpstra test and the Kendall tau correlation for the detection of circadian transcripts. The second is MetaCycle (herein MC) (14), which integrates the *P*-values of JTK and Lomb–Scargle [or LS; another existing algorithm for analyzing rhythmic patterns (15)], using Fisher's meta-analysis approximation. Both methods are non-parametric, and hence have low statistical power. To avoid the scarcity of identified genes with these methods, JTK results are often reported with unadjusted *P*-values and MetaCycle combines the *P*-values obtained using JTK and LS (16,17) using Fisher’s method, despite the problems of combining dependent *P*-values with this method (18)

Here, we have developed CircaN, a natural approach for the detection of circadian-like expression profiles based on fitting circadian curves to the data using non-linear least squares (NLS). Our method fits several curve types to time-series omics data and selects the one with the best fit, providing amplitude, period and phase estimations, along with their *P*-values, and goodness of fit measures. To facilitate the process of identifying circadian genes, we provide a combined corrected *P*-value of the amplitude and period of the curve, which when used along with the goodness-of-fit can be used to divide circadian from other patterns. By analyzing multiple biological and *in silico* datasets, here we find that the performance of circadian-mining algorithms greatly depends on technical and biological characteristics that cannot always be anticipated, such as the amount of noise, or sampling strategy. We show that by combining CircaN with non-parametric models (JTK and MC), we robustly identify almost every circadian gene in a given dataset, with a negligible number of false positives (FPs). We also provide here an R package to ease the implementation of this combined approach, enable optimal identification of circadian genes and promote new discoveries on circadian physiology. Finally, because CircaN is able to accurately determine the main parameters of a circadian curve, our method opens new possibilities to work with circadian genes in a quantitative manner, rather than mere identification of circadian-like patterns.

## MATERIALS AND METHODS

### *In silico* data generation

**Figure 1.**
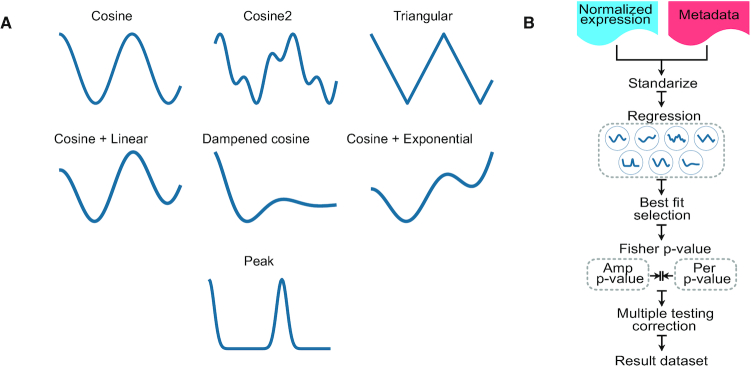
CircaN curve patterns and workflow. (**A**) Curve types included in the CircaN algorithm (19). (**B**) CircaN workflow. Normalized expression and metadata are introduced as parameters in the CircaN function. The data are then standardized and fit to each of the curve types in (A). The curve best fitting the data is selected and a combined *P*-value is computed for the amplitude and period parameters. Finally, multiple testing correction is applied to each *P*-value and the results are returned, including gene lists, parameter estimations and goodness-of-fit measures.

We generated *in silico* values simulating a 24 h long dataset, with sampling once every hour, and with three replicates. The dataset contained a total of 10 000 genes, from which 30% featured circadian oscillations. The non-circadian portion of the genes was generated using a normal distribution with mean and standard deviation randomly obtained from gamma distributions, reproducing the range of amplitudes and variance between replicates and across conditions. The rhythmic features were created using eight different curve patterns, namely, cosine, cosine + outlier, squared cosine, triangular, dampened cosine, cosine + linear, cosine + exponential and peak, following the recommendations previously published (19). Figure 1A shows every shape potentially defined as circadian, except for the cosine + outlier pattern, which consists of a cosine curve with a deliberate random outlier point, and is considered a cosine-type curve. The rhythmic genes were created with periods randomly selected from a uniform distribution between 20 and 28 h, and phases also randomly generated from a half-normal distribution (*θ* = 0.2). To create datasets with different sampling frequencies, we created subsets with the samples corresponding to frequencies of 2, 3 and 4 h.

### *In silico* benchmarking

CircaN, JTK (R package, https://openwetware.org/wiki/HughesLab:JTK_Cycle) and MetaCycle (14) v.1.1.0 were run on the *in silico* data.

Molecules (genes, proteins and metabolites) with a *q*-value < 0.05 were considered rhythmic in JTK and MetaCycle. For CircaN, genes were considered rhythmic if they had a combined *q*-value of the estimated parameters (period and amplitude) below 0.05 and an R-squared ≥ 0.7. These results were then analyzed, and the number of true positives (TP), FP or false negatives was calculated for each algorithm.

### CircaN workflow

For the CircaN analysis we ran the following workflow that can be found at https://github.com/AndreaRP/CircaN. Briefly, first the expression data and the metadata corresponding to the sample (e.g. animal ID), time point and individual of each sample are introduced. The parameters that can be specified in the function include the algorithm for the NLS regression (Port, plinear or Gauss–Newton), the initial value for the period (set to the desired period for the target genes) and maximum and minimum periods to regress. The gene expression (X) of each gene *i* at each time point *t* is standardized as in (1):

(1) $$\begin{equation*}\;{Z_{ti}} = \;\frac{{\left( {{X_{ti\;}} - \;{{\rm{\mu }}_i}} \right)}}{{{S_i}}}\end{equation*}$$

where μ is the average expression of the gene across time, and S its standard deviation. The time is given in hours.

The algorithm fits the data to the wave patterns as detailed in Figure 1B. The initial value for the amplitude is automatically calculated for each gene as the absolute value of half the range of its standardized expression. The initial value for the period can be manually specified; for our benchmarking we set it to 24 h, which is the default for CircaN. Of note, CircaN delivers the phase estimates as the actual phase, not the peak time of expression, which is different from other analytical tools. Next, CircaN calculates the R-squared, the AIC and BIC to assess the goodness of fit. It also computes the adjusted *P*-value for each estimated parameter. After this step, CircaN has seven potential results for each gene, one per theoretical circadian profile, according to (19), and on the next step it selects the fit with the lowest AIC as the best match and the rest are discarded. Finally, CircaN calculates the combined *P*-value of the period and amplitude parameters using Fisher's method (20) and its corresponding adjusted *P*-value by Benjamini–Hochberg Procedure (21). Fisher's procedure merges the probability values of each independent test into one statistic by means of the formula in (2):

(2) $$\begin{equation*}\;X_{2k}^2 = \; - 2\mathop \sum \limits_{i\; = \;1}^k ln\left( {{{\rm{p}}_i}} \right)\;\end{equation*}$$

where p_i_ is the *P*-value of the ith hypothesis test.

### Sample processing for RNA-sequencing and raw data pre-processing

Mice were kept in a normal 12 h light/12 h dark controlled mouse facility and experimental procedures were approved by the Animal Care and Ethics Committee of CNIC and the regional authorities. Whole intact livers were harvested at zeitgeber times (ZT) 1, 5, 9, 13, 17 and 21, for all mice and snap-frozen in 1 ml of TRIzol each. Whole RNA was obtained subsequently using mechanical disruption (Polytron PT 6100; Kinematica) and chloroform extraction and cleaned up using silica-based spin columns (Qiagen) according to manufacturer instructions. RNA quality was checked using capillary electrophoresis (Agilent). Five mice per group were experimented upon, but only the best quality RNA from three of the mice from each group was submitted for whole RNA next generation sequencing in the Genomics Unit of CNIC. A total of 200 ng of RNA was used to generate barcoded RNA sequencing libraries using the NEBNext Ultra RNA Library preparation kit (New England Biolabs). Libraries were sequenced with HiSeq2500 (Illumina) to generate 50-nt single reads, with minimum of 8 million reads per sample. FastQ files for each sample were obtained using CASAVA v1.8 software (Illumina). Reads were further processed using the CASAVA package (Illumina) to demultiplex reads according to adapter indexes and to produce FastQ files. Read quality was determined with the application FastQC. For data analysis, sequencing adaptor contaminations were removed from reads using Cutadapt v1.7.1, and the resulting reads were mapped on the transcriptome (GRCm38 Ensembl gene-build 84) and quantified using RSEM v1.2. Only genes with counts in at least one sample were considered for statistical analysis. The R package limma v3.32.2 was used to normalize the estimated counts from RSEM. Raw data for the RNA sequencing analyses, as well as the normalized counts can be publicly accessed at the Gene Expression Omnibus (GEO; NCBI) with GEO accession no. GSE125867.

### Circadian analyses on biological data

We analyzed data from four different publications (7,11,22,23), and one in-house liver dataset. These data featured different cell types (liver tissue and epidermal stem cells) with varying sampling frequencies and lengths. We performed circadian analyses with CircaN, JTK as a standalone algorithm and MetaCycle 2d, using the combined results of JTK and LS. For all algorithms we established a minimum period of 20 and a maximum of 28. When necessary, we cropped longer datasets to adjust them to a 24 h time frame.

### Gene ontology analysis

All gene ontology (GO) analyses were made using David GO, focusing on the Biological Processes section of the results. We chose to perform a so-called Direct analysis, which provides GO terms that are directly annotated by the source database, without parent terms, and thus reduces the redundancy often found in these type of functional exploration by grouping terms belonging to the same hierarchy family together. For all the gene lists, only terms with an adjusted *P*-value (Benjamini) below 0.05 were considered to be ‘enriched’.

## RESULTS

### Detection of circadian genes using CircaN

CircaN fits a circadian-like curve to the data using NLS. NLS algorithms provide a numerical approximation to obtain the optimum value that minimizes the mean square error in non-linear models for which an analytical solution does not exist (24,25). To encompass the majority of possible expression kinetics featuring true circadian frequencies, we considered seven different oscillatory curve types as templates, namely cosine, cosine square, triangular, cosine plus linear, dampened cosine, cosine plus exponential and peak curves (Figure 1A) (19). CircaN takes the normalized gene expression data as input and performs a standardization to unify the data range. Each gene is then fitted to every curve pattern using NLS optimization to estimate its period, amplitude and phase. If replicates are available, a random effect is included in the model to account for sampling uncertainty, thereby improving the estimation of the parameters of interest, and *P*-values are then calculated for each parameter. Because the rhythmicity of circadian genes is mainly captured by the amplitude and the period parameters, we used Fisher's procedure to combine the *P*-values for both the amplitude and the period, as an overall estimate of each gene's oscillatory behavior. Finally, this new combined *P*-value is corrected for multiple testing using the Benjamini–Hochberg procedure (21). CircaN also provides statistics of the goodness of the fit; for instance, both the Akaike and Bayesian Information Criteria are provided (AIC; BIC, respectively), as well as the R^2^, along with the estimation of the parameters for the curve that best fit the data (Figure 1B). Using the adjusted combined *P*-value and the R^2^ we obtain a reliable list of circadian genes. An additional feature of our NLS model is that it can be used with uneven sampling data, i.e. when the samples have been collected at irregular intervals, or when there are missing time points or replicates.

### CircaN detects circadian genes with larger amplitude and replicate variability

**Figure 2.**
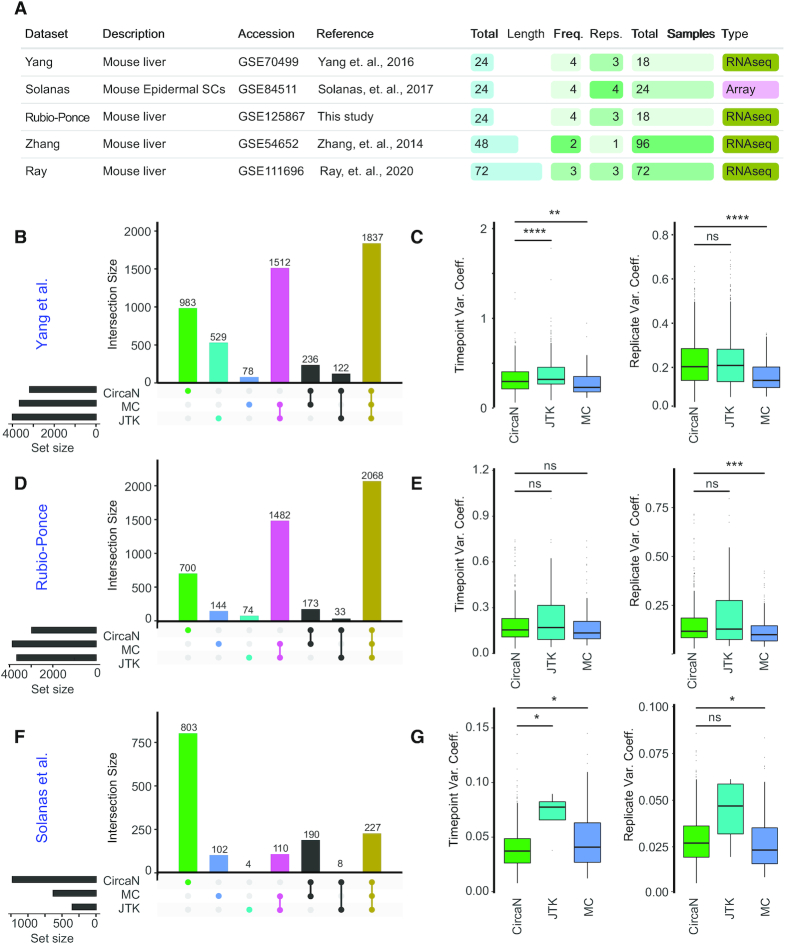
Non-redundant mining of circadian genes across algorithms. (**A**) Table with analyzed dataset configurations. (**B**) Upset plot depicting the concordance of detected genes in the Yang *et al.* dataset (24 × 4 × 3) and (**C**) the variation coefficients between time points and replicates. (**D**) Upset plot depicting the concordance of detected genes in the Rubio-Ponce dataset (24 × 4 × 3) and (**E**) the variation coefficient between time points. (**F**) Upset plot depicting the concordance of detected genes in the Solanas *et al.* dataset (24 × 4 × 3), and (**G**) the variation coefficient between timepoints. ****P < 0.0001, ***P < 0.001, **P < 0.01, *P < 0.05, ns = not significant as determined by a Wilcoxon Test.

In order to test the performance of our NLS algorithm, we used different datasets from publicly available studies, as well as from an in-house circadian RNA sequencing dataset. We selected data with experimental setups as diverse as possible, featuring different total lengths, sampling frequencies, number of replicates and sequencing technologies (Figure 2A), and performed a comparative analysis using CircaN and the two most widely used algorithms in the field, JTK and MC. We initially sought to establish the performance of each algorithm in 24 h-long circadian datasets, which are common in research with mammals (mostly mice), from two different studies (7,22), as well as an in-house dataset of circadian gene expression in the mouse liver. (Figure 2A). We first analyzed a mouse whole liver RNAseq dataset, with sampling every 4 h for 24 h. Analysis of this dataset (Yang *et al.*; (22)) revealed that although many circadian genes were detected by all three algorithms (1837), MC and JTK captured an additional 1512 genes. While this overlap was expected given that JTK is incorporated into the MC model, JTK captured 529 additional genes that were missed by MC. Notably, CircaN further detected unique 983 genes that were undetected by the non-parametric methods (Figure 2B).

We used the results from this first dataset to search for possible causes underlying the differential performance of each method. In particular, we examined the possibility that the identification of circadian patterns in genes was associated with variability of gene expression within and across time points. Variability between time points is a measure of the amplitude, while inter-replicate variability reports technical or biological noise. Hence, we calculated the coefficient of variation (CV) between time points and between replicates for the sets of genes uniquely identified by each algorithm. We found that CircaN and JTK allowed for larger variability between replicates than MC, which in contrast was able to identify circadian genes with smaller amplitudes (Figure 2C). Analysis of a second, in-house generated dataset of the circadian liver transcriptome yielded similar results (Figure 2D): strong coincidence of genes identified by the three methods (2068 genes), a large number of genes shared by MC and JTK (1482), but not CircaN and a noticeable set of genes detected only by CircaN but none of the other two algorithms (700). The characteristics of the genes detected by each method followed the same trend than the previous dataset: CircaN captured genes with larger amplitude and more variability between replicates than MC (Figure 2E). Contrasting with these RNA sequencing analyses, in an additional microarray dataset (Solanas *et al.*; (7)) the number of genes detected by all three algorithms was low (227) (Figure 2F). Notably, in this set CircaN outperformed the other algorithms with 803 unique circadian genes detected, while JTK and MC captured very few specific genes individually (4 and 102, respectively), and 110 genes were detected by both. Once more, the CV across time points was smaller for the genes detected by CircaN than by MC or JTK, but the CV between replicates remained higher than that of the genes detected by MC (Figure 2G). These results might be the consequence of the ‘noisier’ nature of microarray data versus NGS data (26).

**Figure 3.**
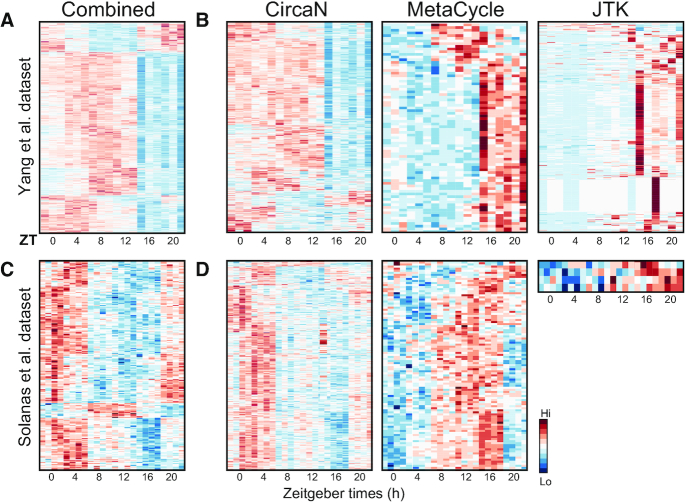
Visualization of common and unique gene profiles detected by each algorithm. (**A**) Heatmap of the genes detected as circadian by all three tested algorithms in the Yang *et al.* dataset. (**B**) Heatmap of the circadian genes detected by CircaN, MetaCycle or JTK, respectively, in the Yang *et al.* dataset. (**C**) Heatmap of the genes detected as circadian by all three algorithms in the Solanas dataset. (**D**) Heatmap of the circadian genes detected by CircaN, MetaCycle or JTK, respectively, in the Solanas dataset. Please see Supplementary Figure 1 for R^2^ values of the detected genes shown here.

Heatmaps of the different collection of genes revealed circadian-like patterns for genes that had been missed by the other algorithms. (Figure 3A and B). Additionally, the R^2^ values of the genes detected by CircaN show the close fit of the genes detected by CircaN to circadian curves. (Supplementary Figure S1a). Thus, the different algorithms identify common as well as distinct gene sets featuring circadian dynamics: CircaN is able to detect rhythmic patterns with a higher degree of noise between replicates, thereby identifying genes with more subtle changes across time. This feature critically allows identification of a substantial number of circadian genes not detected by existing methods, as clearly illustrated in the microarray dataset (Figure 3C and D; Supplementary Figure S1b). MC, in turn, is less permissive with the experimental and biological noise but it is able to detect weaker circadian oscillations.

### CircaN outperforms non-parametric methods in 24-h experiments when biological replicates are limiting

To further assess the performance of each algorithm in different experimental setups, we next analyzed datasets with varying sampling lengths and frequency, as well as number of replicates. In the Zhang dataset (11), which sampled every hour for 48 h (Figure 2A), we found that most oscillating genes were detected by all algorithms (3108 genes), some only captured by CircaN (667), and others were detected only by both MC and JTK (578) (Figure 2A). *In silico* reduction of this data into a 24 h-long experiment, with only one sample every hour, yielded completely different results (Figure 2B). While JTK or MC detected very few genes, CircaN detected over 6800 circadian genes. Similarly, when we analyzed a 72 h-long dataset ((23).; Figure 2C), CircaN detected fewer genes while JTK and MC both captured over 6000 genes. Interestingly, when these data were cropped to a 24-h setting, the results were again similar to the Yang and Rubio-Ponce datasets (Supplementary Figure S2d).

Together, these observations reveal that JTK, MC and CircaN feature strikingly different strengths and weaknesses in identifying *bona fide* circadian genes, depending of the noise, robustness of the circadian pattern and sampling scheme. Since these features cannot be known beforehand, we next examined whether optimal circadian transcript mining could be achieved by the routine combination of non-parametric (MC) and parametric (CircaN) approaches.

### Combination of non-parametric and NLS models optimizes discovery of circadian programs in tissues

**Figure 4.**
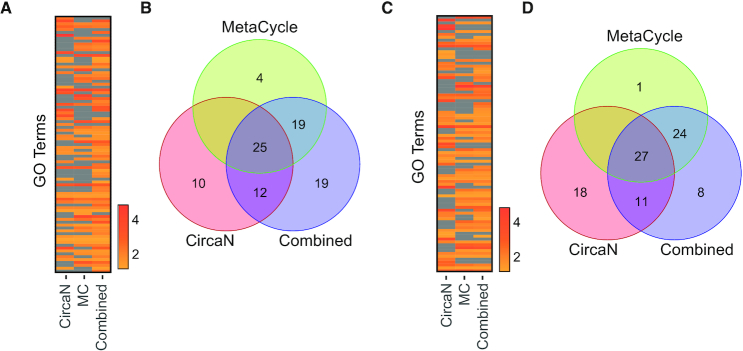
Combination of non-parametric and NLS models improves identification of biological pathways. (**A**) Fold-change in the GO analysis for the terms that were significant enriched (Benjamini < 0.05) in the gene lists of each separate algorithm, and the combined strategy from the Yang *et al.* dataset. (**B**) Venn Diagram shows the overlapping and unique terms found for each algorithm for the Yang dataset. (**C** and**D**) Same analyses as in (A) for the Rubio-Ponce liver dataset. Scales show fold-enrichment.

To assess the robustness of circadian data mining with the different methods, we submitted the gene lists identified by CircaN and MC separately or in combination for pathway analysis. In the Yang *et al.* dataset, the combined list of genes resulted in more biological terms than either of the analyses separately (Figure 4A and B). Moreover, virtually all terms enriched in the combined analysis assembled under relevant categories for liver function, such as lipid metabolism, autophagy, insulin signaling pathway and circadian rhythms (Supplementary Table S1). Similarly, the analysis of the genes detected with the combined strategy for the Rubio-Ponce dataset yielded new functional pathways (GO terms) than either of the separate lists (Figure 4C and D). Again, most terms in the combined list were relevant for liver function (Supplementary Table S2). Thus, combination of MC and CircaN is superior at identifying a substantially higher number of circadian genes and relevant functional pathways.

### Optimal signal-to-noise performance with the combined approach

**Figure 5.**
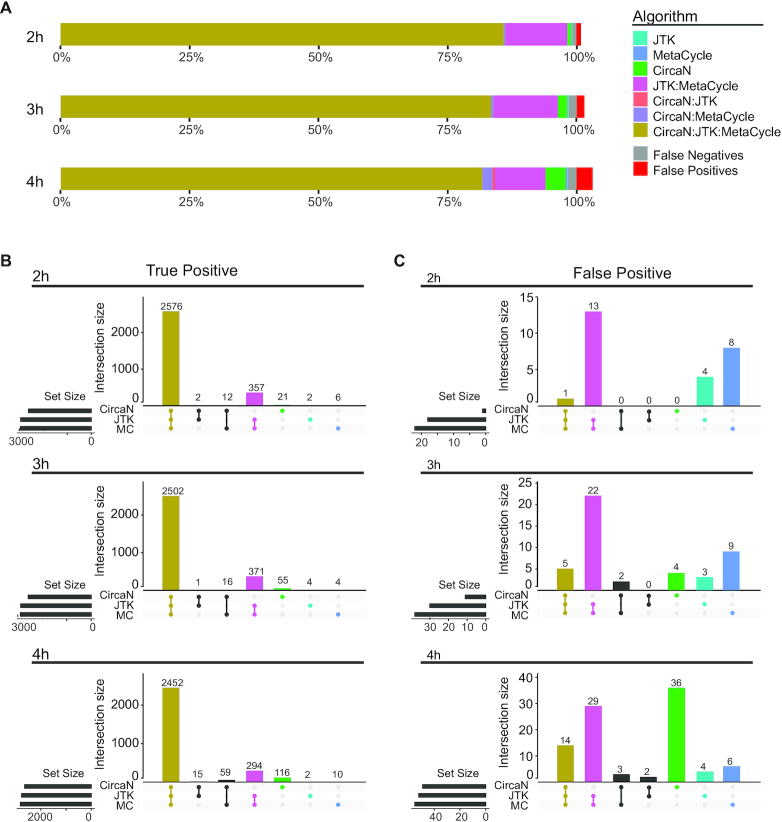
Reduced FP counts using combined modeling. (**A**) Stacked bar plot showing the genes detected in the *in silico* dataset by each algorithm, as well as the amount of FP genes obtained with the combined analysis. Each bar represents a different sampling interval (2, 3 or 4 h) for a 24 h long dataset. (**B**) Upset plots of the TP counts for each algorithm. (**C**) Upset plots of the FP counts for each algorithm.

An obvious caveat of approaches that identify large numbers of genes is that non-circadian events (i.e. FP) can be incorporated and generate biological noise, bias pathway analyses and confound interpretation of the data. To get an estimation of the proportion of FP genes that were introduced by our combined strategy, we built an *in silico* dataset (Supplementary Table S3) in which genes with *bona fide* circadian behavior were pre-determined. We generated a dataset containing 10 000 genes, of which 30% had a type of circadian behavior randomly selected from the different curves shown in Figure 1A, plus a cosine wave with a random outlier point, to assess the performance when there is an outlier in the temporal series. To further reproduce a realistic scenario, transcript values were generated with different variabilities across time points and across replicates in a 24-h experiment, with different sampling frequencies (every 2, 3 and 4 h). Using this approach, we successfully identified virtually all circadian genes from our dataset (98.2–99.2%) and, more importantly, we obtained very low rates of FP gene detection, regardless of the sampling frequency (0.8–3.1%) (Figure 5A–C). Detailed analyses further demonstrated that the majority of *TP* genes were identified by the combination of all three algorithms, while false positivity was more common for individual algorithms, especially at low sampling frequencies (Figure 5C). Thus, by combining strategies that exploit different statistical strengths, we significantly extend the efficient mining of circadian genes in multiple experimental datasets, with almost complete identification of the circadian transcriptome.

## DISCUSSION

Adjusting a non-linear mixed model to the data generated in circadian experiments is, together with time-series analysis, the most natural statistical approach to the detection of circadian patterns from experimental observations. However, economical and ethical concerns and the inherent noise of gene expression data have made non-parametric methods (13) emerge as the tool of reference. Non-parametric tests are known to have less statistical power than parametric ones, and this drawback is typically overcome by using unadjusted *P*-values, especially for microarray data, which usually produce noisier data than NGS (7), or by integrating *P*-values from different methods (14), which represent a relaxation of statistical standards and, more importantly, may introduce biological noise. Here, we have developed a robust computational approach for the identification of circadian genes from transcriptomic data. Our method adds to previously existing algorithms in studies with mammalian models or in other experimental setups in which the available number of individuals is limited, and also when it is necessary or advantageous to use uneven sampled data. Other methods, such as Lomb–Scargle, also offer the possibility to analyze uneven sampling but perform poorly as stand-alone algorithms, particularly in short-term experiments (in-house testing; not shown), whereas commonly used non-parametric models such as JTK do not allow uneven sampling. CircaN analyzes the data generated in gene expression experiments and fits a wide range of curve types, through NLS modeling, to obtain estimates from each fitted curve. It then automatically selects the model that best fits the actual data and presents the corresponding estimation for period, phase and amplitude, along with several metrics regarding the goodness of fit.

The inconsistent performance of the most widely used circadian algorithms on different datasets inspired the development of CircaN. We reasoned that, as a parametric method, it would have the statistical power to detect rhythmic patterns in a broader range of data distributions. Indeed, in our own systematic analysis of several biological datasets, we have found that while MetaCycle is generally able to detect genes with lower amplitudes than either JTK or CircaN, it requires data with low CV across replicates, i.e. a smaller amount of technical and biological noise. When confronted with data of a more variable nature, such as those generated from biological samples (including live animals or patients), the detection of circadian genes using MC drops dramatically. In contrast with these methods, CircaN’s performance is comparatively stable in all the tested datasets. This effect is particularly evident when testing highly variable data, such as microarray-based experiments, where CircaN alone detected 56% of all captured genes. Because the sampling frequency and number of replicates are a strong source of variability, we addressed this important issue by testing several configurations. As expected, *in silico* data show that sampling frequencies closely correlate with overall performance, specifically with the number of FPs detected. Based on our results, we recommend that a minimum of six time points with three replicates be collected for an accurate analysis.

A key conclusion of our study is that the combined use of CircaN and MetaCycle maximizes the detection of circadian genes, without escalating the number of FPs, as shown by analysis of our *in silico* dataset. Importantly, the combined use of non-parametric and NLS-based models optimizes the results and allows a much more stable performance in a wide range of experimental conditions, and should be amenable and simple to use for those interested in dissecting biological patterns in the mammalian transcriptome. This functionality is included into a CircaN R package by means of the *full_mode_analysis* function, which runs all three methods and combines the results into a single file. CircaN is freely available to the community as an R package (https://github.com/AndreaRP/CircaN).

Although we have only tested CircaN with transcriptomics data here, our method should be equally useful for other types of omics data, such as proteomics or epigenomics datasets that display similar circadian variations in quantifiable parameters (protein or chromatin/DNA modifications). Thus, CircaN, alone or in combination with non-parametric algorithms, provides an extremely valuable tool for the identification of circadian parameters under real-life situations, in which the number of samples is typically limited, without significant loss of biological information.

## DATA AVAILABILITY

Raw data for the RNA sequencing analyses as well as the normalized counts can be publicly accessed at the Gene Expression Omnibus (GEO; NCBI) with GEO accession no. GSE125867.

## Supplementary Material

lqab031_Supplemental_Files

## Notes

Present address: Andrés Hidalgo, Area of Cell and Developmental Biology, Centro Nacional de Investigaciones Cardiovasculares, Madrid, 28029, Spain.

Present address: Fátima Sánchez-Cabo, Bioinformatics Unit, Centro Nacional de Investigaciones Cardiovasculares, Madrid, 28029, Spain.
