# Supplementary material for: Combined statistical modeling enables accurate mining of circadian transcription
Source: NAR Genom Bioinform. 2021 Apr 26;3(2):lqab031. doi: 10.1093/nargab/lqab031 (PMC8074341; doi:10.1093/nargab/lqab031)
Supplement: lqab031_Supplemental_Files [file lqab031_supplemental_files.zip › FigS1.pdf]

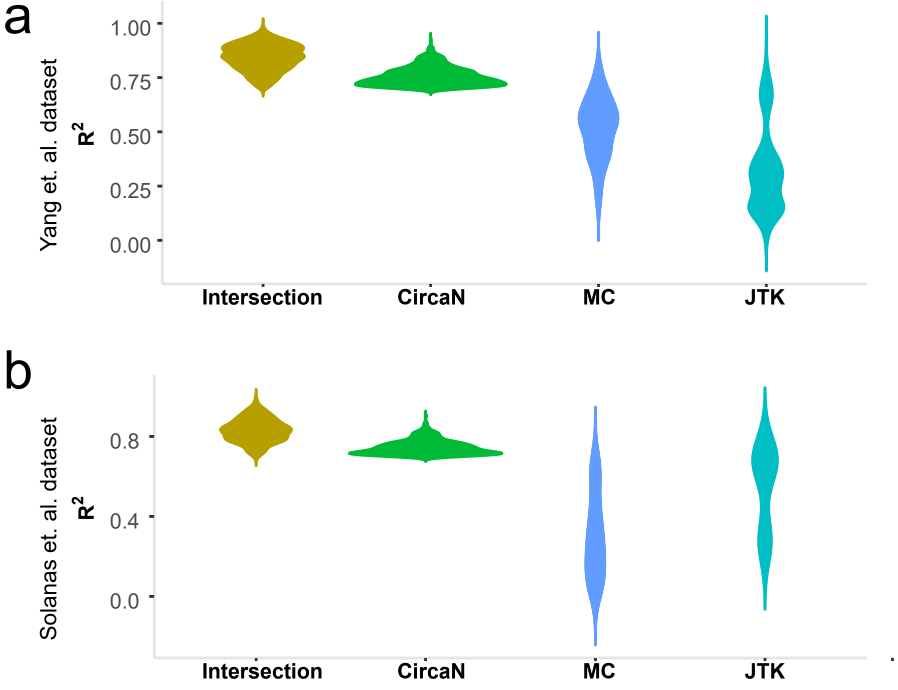
a
Yang et. al. dataset
R2
1.00
0.75
0.50
0.25
0.00
Intersection
CircaN
MC
JTK
b
Solanas et. al. dataset
R2
0.8
0.4
0.0
Intersection
CircaN
MC
JTK
