# Supplementary material for: Combined statistical modeling enables accurate mining of circadian transcription
Source: NAR Genom Bioinform. 2021 Apr 26;3(2):lqab031. doi: 10.1093/nargab/lqab031 (PMC8074341; doi:10.1093/nargab/lqab031)
Supplement: lqab031_Supplemental_Files [file lqab031_supplemental_files.zip › Suppl. Information_Rubio2021.pdf]

Supplementary Information

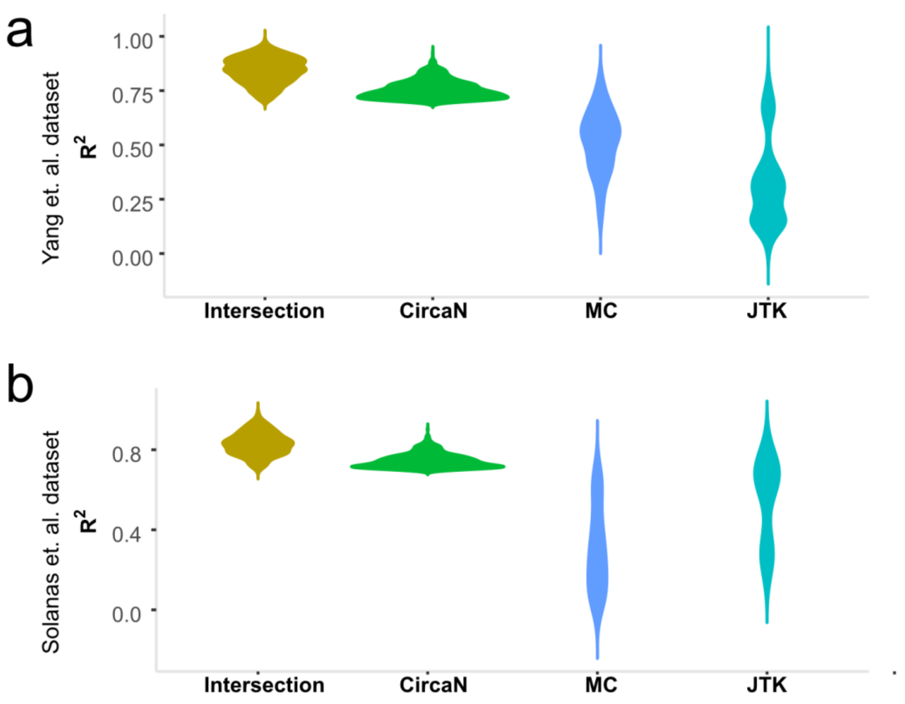

Supplementary Figure 1. R^2 values of the detected genes. (A) R^2 values for each of the sets of genes detected by all three algorithms (Intersection), CircaN, MetaCycle (MC) and JTK, in the Yang dataset shown as heatmap in Figure 3. **(B)** R^2 values for each of the sets of genes detected by all three algorithms (Intersection), CircaN, MetaCycle (MC) and JTK, in the Solanas dataset, shown as heatmap in Figure 3.

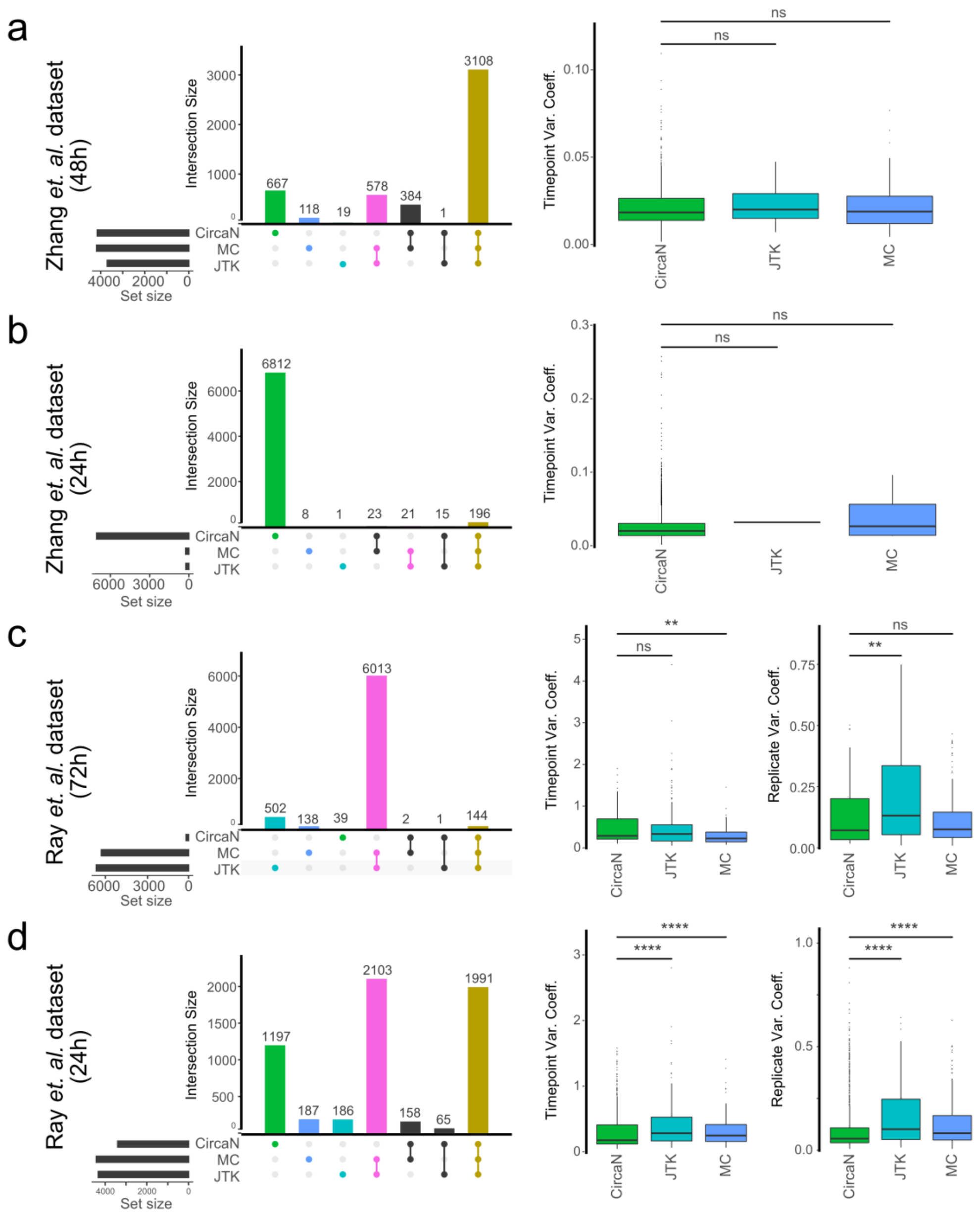

Supplementary Figure 2. Non-redundant mining of circadian genes across algorithms (extended). (A) Upset plot depicting the concordance of detected genes in the Zhang dataset (48x1x1) on the left, and the variation coefficient between timepoints and replicates. **(B)** Upset plot depicting the concordance of detected genes in the Zhang dataset (24x1x1) on the left, and the variation coefficient between timepoints and replicates. **(C)** Upset plot depicting the concordance of detected genes in the Ray dataset (72x4x3) on the left, and the variation coefficient between timepoints and replicates. **(D)** Upset plot depicting the concordance of detected genes in the Ray dataset (24x3x3) on the left, and the variation coefficient between timepoints and replicates. ****p<0.0001, ***p<0.001, **p<0.01, *p<0.05, ns=not significant as determined by a Wilcoxon Test.

Supplementary Tables

Supplementary Table 1. Yang dataset GO Terms file. Go terms analysis of the MC and CircaN combined results from the Yang et al. dataset.

Supplementary Table 2. Rubio-Ponce dataset GO Terms file. Go terms analysis of the MC and CircaN combined results from the Rubio-Ponce et al. dataset.

Supplementary Table 3. *In-silico* dataset. Full *in-silico* dataset used for benchmarking.
