# Supplementary figures and images for: Combined statistical modeling enables accurate mining of circadian transcription
Source: NAR Genom Bioinform. 2021 Apr 26;3(2):lqab031. doi: 10.1093/nargab/lqab031 (PMC8074341; doi:10.1093/nargab/lqab031)

**a**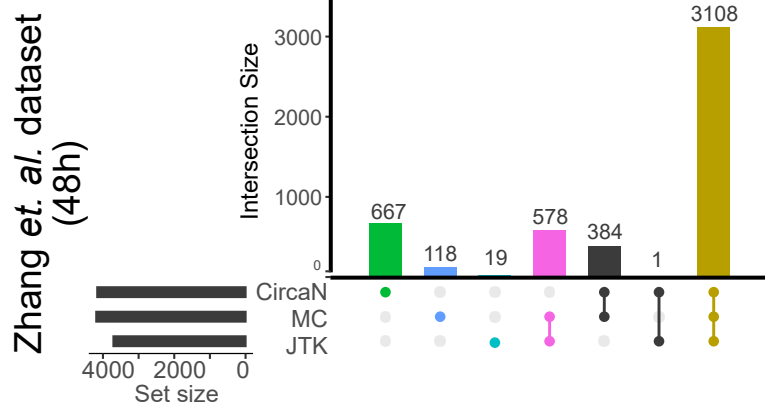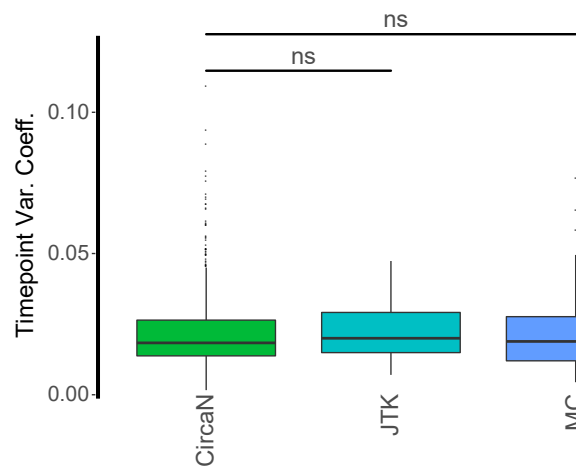**b**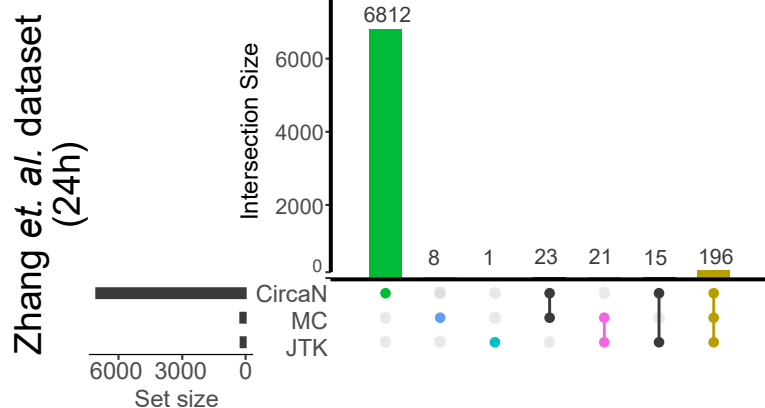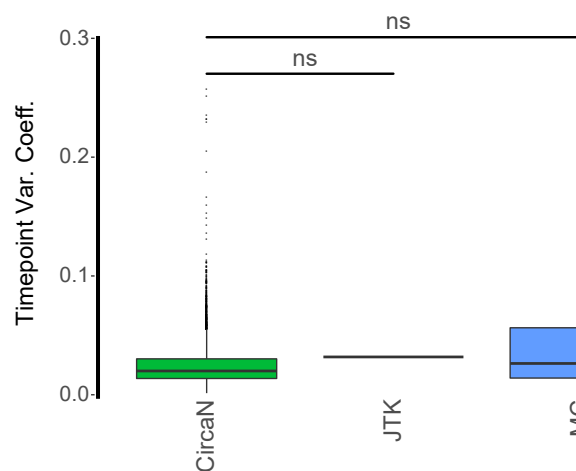**c**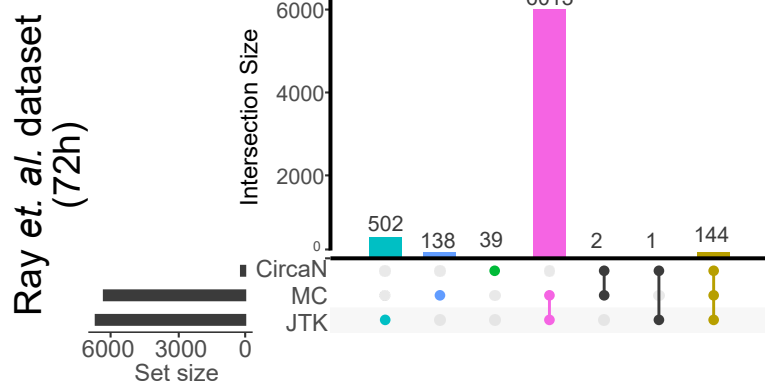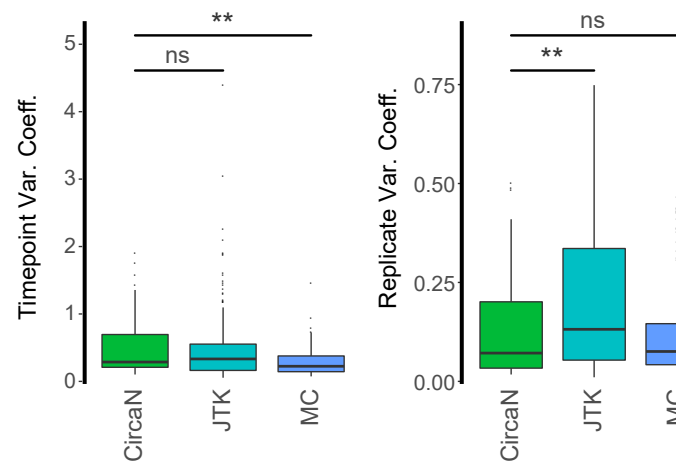**d**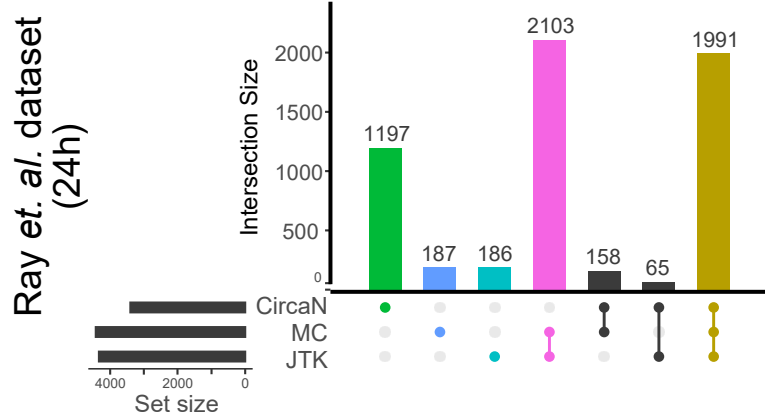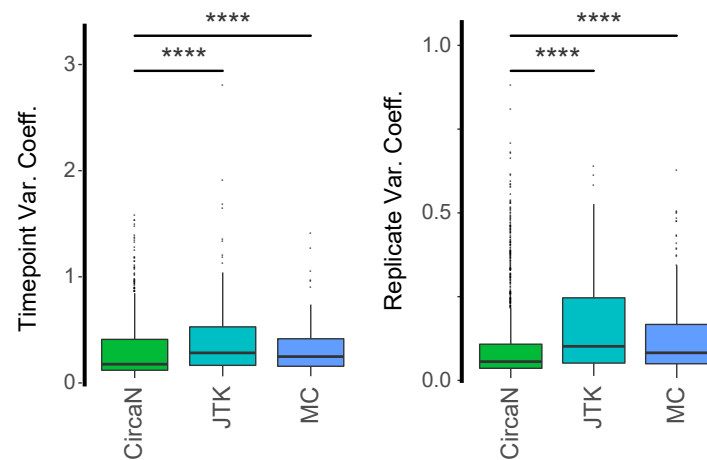

Supplement: lqab031_Supplemental_Files [file lqab031_supplemental_files.zip › FigS2.pdf]
